# Building Researchers' Capacity for Embracing the Engagement of Older Adults in Research

**DOI:** 10.1093/geroni/igab046.576

**Published:** 2021-12-17

**Authors:** Rebecca Berman, Rachel Lessem, Margaret Danilovich

**Affiliations:** 1 Leonard Schanfield Research Institute, CJE SeniorLife, Illinois, United States; 2 CJE SeniorLife, Chicago, Illinois, United States; 3 CJE SeniorLife, Skokie, Illinois, United States

## Abstract

The Sage Resource Project aimed to broaden the pool of researchers who include the voice of older adults using long-term services and supports (LTSS) in research processes. We developed training to build researcher capacity to engage older adults through the development of Sage Model research advisory boards. Methods included training strategies for learning mode, design, duration, and emphasis of content that were informed by results of a researcher needs assessment and input from 2 older adult research advisory boards. Over 100 researchers registered for a 4-webinar series. All respondents to webinar evaluations (22) reported learning about topics that aligned with webinar objectives and had interest in engaging older adult stakeholders and/or developing an older adult research advisory board in the future. Representatives from five universities expressed interest attending online interactive workshops to build advisory boards. Lessons learned identify directions for research on best practices for developing older adult advisory groups.

